# Retraction: Endoplasmic reticulum stress promotes autophagy and apoptosis and reverses chemoresistance in human ovarian cancer cells

**DOI:** 10.18632/oncotarget.28801

**Published:** 2025-12-31

**Authors:** Jin-Long Hu, Xin-Long Hu, Ai-Ye Guo, Chao-Jie Wang, Yi-Yang Wen, Shun-Dong Cang

**Affiliations:** ^1^Department of Oncology, Henan Provincial People’s Hospital, Zhengzhou 450000, P. R. China; ^2^Department of Medical Imaging Technology, Henan University of Chinese Medicine, Zhengzhou 450000, P. R. China; ^3^Laboratory of Clinical Research, Henan Provincial People’s Hospital, Zhengzhou 450000, P. R. China

**This article has been retracted:** Oncotarget has completed its investigation of the article, which uncovered multiple instances of image duplication and overlap. Specifically, Figure 2B contains western blot bands also found in Figure 9A of [1], Figure 5A of [2], and Figure 7C of [3]. The b-actin blots in Figures 5B and 6A appeared as different blots in papers [4, 5], respectively. Multiple internal and external duplications of plate images from the colony formation assay were found in Figures 3D, 6D and 8D. The Figure 8D images were also found in Figure 2C of [6], in Figure 4B of [7], in Figure 5C of [8], and in Figure 7C of [9]. In addition, overlaps were found in the MDC staining fluorescent images across Figures 3E and 6E, and 6E and 8E. The authors have failed to respond to repeated requests for documentation to address these concerns. In light of these facts, the Editorial decision has been made to retract this paper.

Original article: Oncotarget. 2017; 8:49380–49394. 49380-49394. https://doi.org/10.18632/oncotarget.17673
